# Distinct Activities of Tfap2A and Tfap2B in the Specification of GABAergic Interneurons in the Developing Cerebellum

**DOI:** 10.3389/fnmol.2017.00281

**Published:** 2017-08-31

**Authors:** Norliyana Zainolabidin, Sandhya P. Kamath, Ayesha R. Thanawalla, Albert I. Chen

**Affiliations:** ^1^School of Biological Sciences, Nanyang Technological University (NTU) Singapore, Singapore; ^2^School of Life Sciences, University of Warwick Coventry, United Kingdom; ^3^A*STAR, Institute of Molecular and Cell Biology Singapore, Singapore

**Keywords:** Tfap2A, Tfap2B, cerebellum, GABAergic interneurons, transcription factors, development

## Abstract

GABAergic inhibitory neurons in the cerebellum are subdivided into Purkinje cells and distinct subtypes of interneurons from the same pool of progenitors, but the determinants of this diversification process are not well defined. To explore the transcriptional regulation of the development of cerebellar inhibitory neurons, we examined the role of Tfap2A and Tfap2B in the specification of GABAergic neuronal subtypes in mice. We show that Tfap2A and Tfap2B are expressed in inhibitory precursors during embryonic development and that their expression persists into adulthood. The onset of their expression follows Ptf1a and Olig2, key determinants of GABAergic neuronal fate in the cerebellum; and, their expression precedes Pax2, an interneuron-specific factor. Tfap2A is expressed by all GABAergic neurons, whereas Tfap2B is selectively expressed by interneurons. Genetic manipulation via *in utero* electroporation (IUE) reveals that Tfap2B is necessary for interneuron specification and is capable of suppressing the generation of excitatory cells. Tfap2A, but not Tfap2B, is capable of inducing the generation of interneurons when misexpressed in the ventricular neuroepithelium. Together, our results demonstrate that the differential expression of Tfap2A and Tfap2B defines subtypes of GABAergic neurons and plays specific, but complementary roles in the specification of interneurons in the developing cerebellum.

## Introduction

The cerebellum plays a critical role in sensory-motor integration and is important for the precise coordination of body, limb and eye movements as well as adaptation and learning of motor skills (Eccles et al., 1967; Thach and Bastian, 2004; Ito, 2006). The ability of the cerebellum to carry out these tasks relies on the specification of distinct classes of cerebellar neurons and the assembly of these neurons into functional circuits during development. Despite consisting of less than 10 percent of all neurons in the cerebellum (Andersen et al., 1992; Korbo et al., 1993), the different subtypes of inhibitory neurons play important roles in cerebellar function by providing feedback and feedforward inhibition (Eccles et al., 1967; Ito, 2006). Cerebellar cortical inhibitory neurons use gamma-aminobutyric acid (GABA) and/or glycine and are made up of four major subtypes: stellate, basket, Purkinje and Golgi cells (Miale and Sidman, 1961; Sillitoe and Joyner, 2007). Stellate and basket cells provide feedforward inhibition to Purkinje cells and together regulate motor learning and adaptation as well as fine motor movements (Eccles et al., 1967; De Zeeuw et al., 1998; Wulff et al., 2009; Heiney et al., 2014). Golgi cells, on the other hand, provide feedback inhibition to granule cells to regulate motor coordination and compound movement (Watanabe et al., 1998; D’Angelo et al., 2013). Even though the electrophysiological and morphological properties as well as, to some extent, functional relevance of cerebellar inhibitory neurons have been examined (Palay and Chan-Palay, 1974; Ito, 2006), the molecular mechanisms underlying the diversification of distinct subtypes of cerebellar inhibitory neurons are not well defined.

There is growing evidence that the specification of inhibitory neuronal identity in the cerebellum is controlled by the expression of a set of transcription factors (Pascual et al., 2007; Zordan et al., 2008; Hori and Hoshino, 2012; Hoshino, 2012; Leto and Rossi, 2012). GABAergic neurons of the cerebellar cortex originate from the ventricular neuroepithelium and express basic helix-loop-helix (bHLH) transcription factor Ptf1a, which is required for generation of stellate, basket, Purkinje and Golgi cells (Hoshino et al., 2005; Pascual et al., 2007; Yamada et al., 2014). GABAergic projection neurons, the Purkinje cells, are generated by progenitors within the cerebellar ventricular zone (VZ) and require Corl2/Skor2 for differentiation (Minaki et al., 2008; Nakatani et al., 2014). Another bHLH transcription factor, Olig2, is expressed in the embryonic VZ, and some Olig2-expressing cells differentiate into Purkinje cells (Seto et al., 2014b; Ju et al., 2016). GABAergic interneurons in the cerebellar cortex, which include stellate, basket and Golgi cells, and in the deep cerebellar nuclei (DCN), are generated in the same region and express Pax2 as GABAergic interneuron precursors that proliferate in the white matter (WM) layer (Maricich and Herrup, 1999; Leto et al., 2006, 2009; Weisheit et al., 2006). A set of homeobox transcription factors Lhx1/5 is expressed by all GABAergic postmitotic neurons, however, deletion of Lhx1 and Lhx5 results only in the loss of Purkinje cells, but not GABAergic interneurons (Zhao et al., 2007). Even though a transcriptional program for cerebellar GABAergic neuronal specification is emerging, whether differentially expressed transcription factors have the capability to specify GABAergic interneurons has not yet been identified.

Transcriptome analysis has revealed a number of transcription factors that are regulated by Ptf1a including Lhx1, Lhx5, Corl2, Ngn2 and Pax2 (Borromeo et al., 2014; Russ et al., 2015). Also included in this list are two members of the Transcription Factor AP-2 (Tfap2) family of proteins Tfap2A and Tfap2B (Meredith et al., 2013; Borromeo et al., 2014; Jin et al., 2015; Russ et al., 2015). Tfap2A and Tfap2B have been demonstrated to play important roles for the development of a variety of tissues including the nervous system, kidney, skeleton, skin, limbs and the eye (Schorle et al., 1996; Zhang et al., 1996; Nottoli et al., 1998; West-Mays et al., 1999; Eckert et al., 2005; Seberg et al., 2017). For instance, in the developing nervous system, Tfap2A and Tfap2B are required for the survival of sympathetic and sensory ganglia progenitors (Schmidt et al., 2011). In the developing mouse retina, Tfap2A and Tfap2B have been shown to promote the differentiation of GABAergic and glycinergic amacrine cells (Jin et al., 2015). Thus, these two members of the Tfap2 family of transcription factors appear to regulate proliferation and survival of various cell types and may also be involved in the specification of distinct neuronal subtypes during development.

In addition to the sympathetic and sensory ganglia, both *Tfap2A* and *Tfap2B* transcripts are expressed in the developing and mature mouse cerebellum (Moser et al., 1995, 1997; Shimada et al., 1999), but the neuronal subtypes within the cerebellum that express these transcription factors have not yet been determined. Moreover, the functional significance of these transcription factors in the specification of cerebellar neuronal subtypes is not known. In this study, we examine the expression pattern and function of Tfap2A and Tfap2B in the mouse cerebellum. First, we assessed the spatial-temporal expression of Tfap2A and Tfap2B across embryonic and postnatal stages. Next, we compared their expression with cell type-specific and developmental markers. Finally, using *in utero* electroporation (IUE), we explored the functional significance of Tfap2A and Tfap2B during the development of cerebellar GABAergic neuronal subtypes.

## Materials and Methods

### Animals

C57BL/6JInv mice were used in this study. All mice were housed and bred in Agency for Science, Technology and Research Biological Resource Centre on a 12 h light/dark cycle with free access to food and water. This study was carried out in accordance with the recommendations of Agency for Science, Technology and Research Biological Resource Centre. All protocols were approved by the Institute of Animal Care and Use Committee in accordance with the National Advisory Committee for Laboratory Animal Research guidelines.

### Immunohistochemistry

Mice were anesthetized with 2.5% Avertin [0.025 g/mL 2,2-2 Tribomoethanol (Sigma) in 2-Methyl-2-butanol (Sigma)] prior to perfusion with 0.9% saline and 4% paraformaldehyde (Sigma). Early postnatal tissues were then post-fixed for 1 h while adult tissues were post-fixed overnight in 4% paraformaldehyde. Pregnant mice were euthanized using carbon dioxide gas chamber and confirmed through cervical dislocation. Embryos were collected in 4% paraformaldehyde and post-fixed for 30 min. All tissues were preserved overnight in 30% sucrose (1st BASE) and stored in −80°C in O.C.T compound (Sakura Finetek). All tissues were sectioned at 16–20 μm using a cryostat (ThermoScientific).

Tissue sections were permeabilized with 0.3% Triton-X (OmniPur) in phosphate buffered saline (PBS; 1st Base) for 10 min and blocked for 1 h with 2% horse serum (Invitrogen) and 0.1% Triton-X in PBS. The sections were then incubated with primary antibodies overnight in 4°C. On the following day, the tissues were washed with 0.1% Triton-X in PBS for 3 min three times and incubated with secondary antibodies for 1 h. Tissue sections were again washed and stained with DAPI before being mounted with a coverslip.

Primary antibodies used were as follows: rabbit anti-Tfap2B (1:10,000; gift from T. Jessell, Columbia University), rabbit anti-Ptf1a (1:1000; gift from C. Wright, Vanderbilt University), rabbit anti-Tfap2A (1:500; Santa Cruz Bio Technology), goat anti-Pax6 (1:500; Santa Cruz Bio Technology) and mouse anti-Pax2 (1:500; Santa Cruz Bio Technology), mouse anti-Tfap2A (2 μg/mL; developed by T. Williams, Developmental Studies Hybridoma Bank) and mouse anti-Lhx1/5 (2 μg/mL; developed by T. Jessell, Developmental Studies Hybridoma Bank), goat anti-Olig2 (1:250; R&D), mouse anti-Calbindin (1:5000; Swant) and goat anti-Parvalbumin (1:5000; Swant), rat anti-green fluorescent protein (GFP) (1:1000; Nacalai Tesque) and rat anti-RFP (1:500; Chromotek). Secondary antibodies used were as follows: Donkey anti-Rabbit Alexa Fluor 488, Donkey anti-Rabbit Alexa Fluor 647, Donkey anti-Goat Alexa Fluor 555, Donkey anti-Mouse Alexa Fluor 647, Donkey anti-Rat Alexa Fluor 488 and Donkey anti-Rat Alexa Fluor 555 (1:1000; Molecular Probes).

### Generation of Expression Constructs

For overexpression (OE) studies, pCDH lentivector (p*EF1*-MCS-IRES-RFP) was used (System Bioscience, #CD531A-2). Individual transfer vectors containing open reading frames of *Tfap2A* and *Tfap2B* (GeneArt Invitrogen) were sub-cloned into multiple cloning site (MCS) of pCDH lentivector. Plasmids were digested with *Xba*I and *Not*I (New England Biolabs) restriction enzymes and isolated on 1% agarose gel. Digested plasmids were ligated using T7 DNA ligase (New England Biolabs) for 20–22 h at 16°C. For knockdown studies, pLL3.7 lentivector (p*U6*-MCS-*CMV*-GFP; Addgene) was used to express target short hairpin RNA (shRNA) under *U6* promoter and GFP reporter controlled by *cytomegalovirus* (*CMV*) promoter. DNA oligonucleotides which will be transcribed into shRNA were designed using Invitrogen BLOCK-iT™ RNAi Designer. It comprised of *Xho*I overhang (5′ TCGA 3′) on the 5′ end of the antisense strand and a short hairpin loop (5′ TTCAAGAGA 3′). Targeting sequence used for scramble is 5′ TGCCCTACCACCGAGGTCAA 3′, for Tfap2A knockdown is 5′ TGACAACATTCCGATCCCAATG 3′ and for Tfap2B knockdown is 5′ TGCATGGACAAGATGTTCTTGA 3′. Forty micromolar of sense and antisense oligonucleotides diluted in 1X T4 DNA ligase buffer (New England Biolabs) were annealed by boiling at 100°C for 10 min and cooled to room temperature. Subsequently, both the pLL3.7 and annealed oligonucleotides were digested, separately, with *Xho*I and *Hpa*I (New England Biolabs). Ligation was carried out similar to the OE construct. Cloned vectors were sequenced by Institute of Molecular and Cellular Biology, DNA Sequencing Facility.

### Western Blot

Human embryonic kidney 293T (HEK-293T) cells were seeded at a density of 500,000 cells per well in a 6-well tissue culture plate a day before transfection. OPTI-MEM reduced serum media (Gibco) was used to dilute 1 μg of DNA as well as Lipofectamine 2000 reagent (Invitrogen). Lipid-DNA mixture was introduced into the cells and was incubated for 48 h before protein isolation.

Cells were washed once with PBS before proteins were isolated using M-PER mammalian protein extraction reagent (Pierce) which is supplemented with phosphatase inhibitor tablet (Pierce) and protease inhibitor cocktail (Sigma). Proteins were subsequently quantified via colorimetric assay (Bio-Rad) according to manufacturer’s protocol. Twenty microgram of proteins were used for fractionation by SDS-PAGE and transferred to a PVDF membrane using iBlot^®^ Dry Blotting System (Invitrogen). After incubation with 2% Bovine Serum Albumin (BSA) diluted in TBS-T (10 mM Tris [pH 8.0], 150 mM NaCl, 0.5% Tween 20) for an hour, this blocking buffer was decanted and primary antibodies were incubated overnight at 4°C.

The primary antibodies used were as follows: rabbit anti-Tfap2A (1:500; Santa Cruz Bio Technology), rabbit anti-Tfap2B (1:500; Santa Cruz Bio Technology), and mouse anti-GAPDH (0.05 μg/mL; Sigma). On the next day, membranes were washed with TBS-T thrice, for 10 min each before being incubated with appropriate secondary antibodies. The secondary antibodies used were diluted in blocking buffer as follows: anti-mouse IgG fragment-peroxidase antibody produced in goat (1:3000; Sigma) and anti-rabbit IgG fragment-peroxidase antibody produced in goat (1:3000; Sigma). Membrane was washed again, similarly, before being developed with the ECL system (Cell Signaling) according to manufacturer’s protocol.

### *In Utero* Electroporation

C57BL/6J pregnant mice were anesthetized with 2% isoflurane throughout the procedure and were kept warm using a homeothermic blanket. Hair on the abdomen area was shaved and skin cleaned with cotton swabs soaked in 70% ethanol. A small incision (5–10 mm) on the abdomen through the midline was done and gauze moistened with 42°C 0.9% saline was placed around the incision. Another incision on the muscle was made and both of the uterine horns were carefully placed on the gauze. From time to time, the exposed embryos were moistened with warm saline. 2–5 μL of purified plasmids (1 μg/μL) with Fastgreen Dye (0.1 mg/mL) were delivered into the fourth ventricle using a glass capillary. Electrodes were positioned in between the ventricles and five pulses of 250 mV for 50 ms each, were delivered. Uterine horns were returned into the peritoneal cavity and warm saline was added before the lining of cavity and the skin was sutured. Mice were given Meloxicam for post-operative pain management.

### Image and Cell Count Analysis

Confocal images from immunohistochemistry were captured using Zeiss LSM-710 Confocal Microscope System (Axio Imager Z2 Stand) on ZEN2011. All images were processed with ImageJ (National Institute of Health).

For the Tfap2-Pax2 cell count analysis, two sagittal sections were obtained from the vermis region of each of the three brains and cells were counted, Using ImageJ, specific regions of interest were manually drawn around all nuclei positive for Tfap2A, Tfap2B and Pax2 and were subsequently scored. Immuno-positive neurons were then grouped and percentages were calculated.

Analysis for *in utero* electroporation experiments was assessed by counting all, transfected cells in the WM layer from multiple sagittal sections (approximately 10–25 sections, 100–500 cells) were counted for three brains. Cells were scored for the respective molecular markers and percentages were calculated. Experimental groups were compared against control groups.

### Statistical Analysis

A non-parametric student’s *t*-test was carried out in all the experiments that require statistical analysis. Data represents mean ± SEM. All data was analyzed with GraphPad Prism 6. The statistical data are described in each figure legend.

## Results

### Analysis of the Spatial Expression Pattern of Tfap2A and Tfap2B in the Developing and Mature Cerebellum

**Figure 1:**
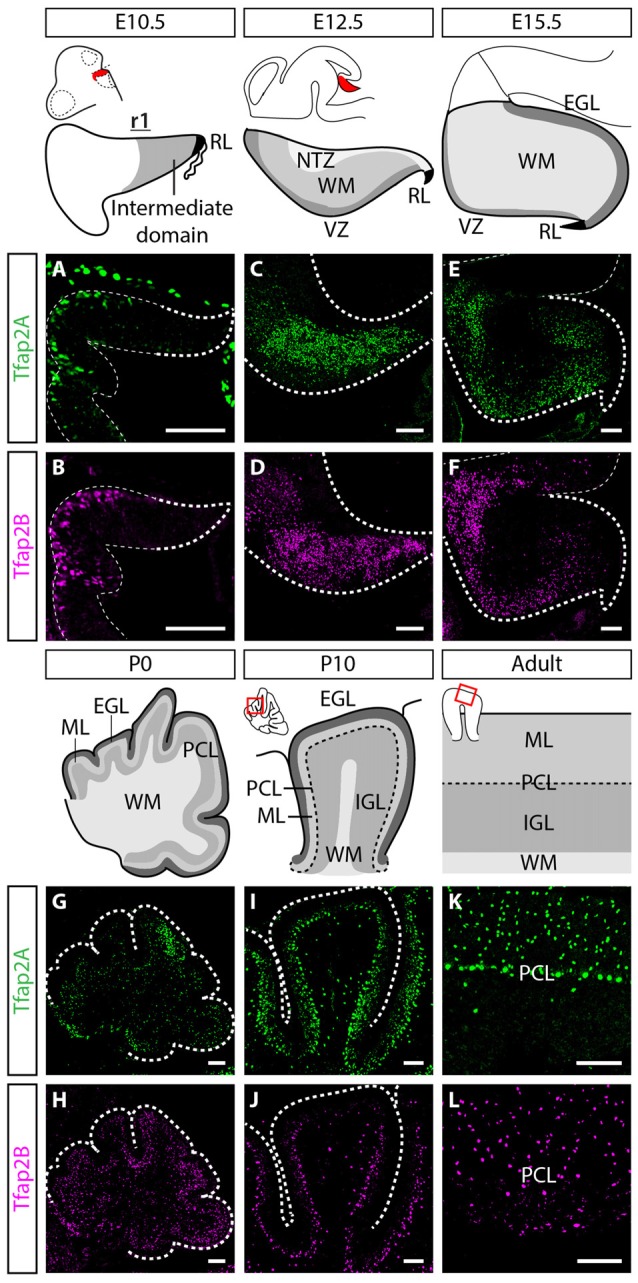
Spatiotemporal expression pattern of Tfap2A and Tfap2B in the cerebellum. **(A,B)** Expression of both Tfap2A (green, **A**) and Tfap2B (magenta, **B**) is not detected in the cerebellar derivatives in r1 at E10.5. **(C–H)** Expression of Tfap2A and Tfap2B is detected in the underlying WM at E12.5 **(C,D)** and E15.5 **(E,F)** where inhibitory neurons transit after exiting the VZ. At early postnatal stages, P0 and P10, Tfap2A, but not Tfap2B, is expressed in the PCL **(G,I)**. In addition, by P10 both Tfap2A^+^ **(I)** and Tfap2B^+^ cells **(J)** have entered the lower half of the ML and the IGL. **(K,L)** In the mature cerebellum at P150, Tfap2A expression **(K)** is detected in the ML, PCL and IGL whereas Tfap2B expression **(L)** is restricted to the ML and IGL. Analysis performed on cerebellar vermal region of sagittal sections. Abbreviations: EGL, external germinal layer; IGL, internal granular layer; ML, molecular layer; NTZ, nuclear transitory zone; PCL, Purkinje cell layer; RL, rhombic lip; r1, rhombomere 1; VZ, ventricular zone; WM, white matter. Scale bar = 100 μm.

In order to uncover the involvement of Tfap2 transcription factors in the development of GABAergic neurons and determine their functional relevance, we analyzed the expression of Tfap2A and Tfap2B in the developing mouse cerebellum. We first assessed whether Tfap2A and Tfap2B are expressed in the rhombic lip (RL) and the intermediate domain of rhombomere (r)1, where the first cerebellar neurons such as glutamatergic DCN neurons and GABAergic Purkinje cells are generated at embryonic day (E) 10.5 (Hoshino et al., 2005; Machold and Fishell, 2005; Wang et al., 2005). The expression of both Tfap2A and Tfap2B was not detected in either of these regions at this stage (Figures 1A,B). At E12.5, we observed expression of Tfap2A and Tfap2B in the WM layer of the cerebellum, located superior to the VZ where GABAergic neurons are produced (Figures 1C,D; Hoshino et al., 2005). The expression of Tfap2B appears to be more widespread than Tfap2A at this stage which may reflect the expression of Tfap2B in non-cerebellar cell types (Figures 1C–F). Since GABAergic neurons transit in the WM before they reassemble to specific layers in the cerebellar cortex (Zhang and Goldman, 1996; Maricich and Herrup, 1999; Leto et al., 2009), our results suggest that Tfap2A^+^ and Tfap2B^+^ cells partake a GABAergic neuron migratory pathway and are expressed by GABAergic precursors in the embryonic cerebellum.

**Figure 2:**
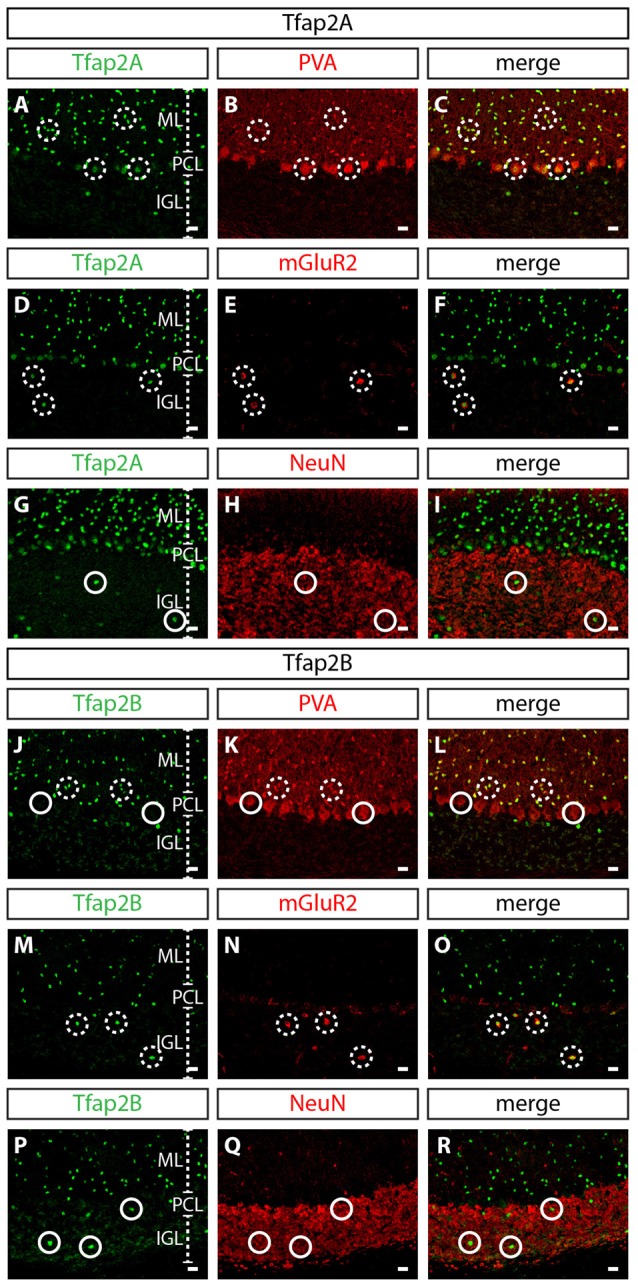
Expression of Tfap2A and Tfap2B is restricted to GABAergic neuronal subtypes in the adult cerebellum. **(A–R)** Analysis of the expression of Tfap2A and Tfap2B with cell type-specific molecular markers in the adult cerebellar cortex. At P60, Tfap2A (green) is coexpressed with PVA (red) in the ML and PCL **(A–C)** and with mGluR2 (red) in the IGL **(D–F)**. Tfap2B (green) is coexpressed with PVA (red) only observed in the ML **(J–L)** and with mGluR2 (red) in the IGL **(M–O)**. No Tfap2A **(G–I)** or Tfap2B **(P–R)** expression (green) is detected in NeuN^+^ granule cells (red) in the IGL. Analysis performed on cerebellar vermal region of sagittal sections. Broken circles indicate cells with colocalized expression, continuous circles indicate no colocalization. Abbreviations: IGL, internal granular layer; ML, molecular layer; PCL, Purkinje cell layer. Scale bar = 10 μm.

To determine whether the expression of Tfap2A and Tfap2B is maintained during late embryogenesis through postnatal stages, we analyzed their expression in perinatal and postnatal cerebellar tissues. The spatial expression pattern of Tfap2A and Tfap2B at E15.5 and P0 supports our earlier observations that Tfap2A and Tfap2B are expressed by GABAergic neurons (Figures 1E–H). In P10 and adult cerebellum, we found Tfap2A^+^ neurons in the molecular layer (ML), Purkinje cell layer (PCL) and internal granular layer (IGL; Figures 1I,K). Tfap2A is expressed by a subset of calbindin^+^ Purkinje cells in the developing cerebellum suggesting that it could mark early-onset Purkinje cell clusters which disperse after birth (Figure 1G, Supplementary Figures S1A–D). However, colocalization analysis in the coronal orientation in the adult cerebellum revealed that Tfap2A is uniformly expressed in parvalbumin^+^ Purkinje cells (Supplementary Figures S1E–H). These results indicate Tfap2A is expressed in a subset of Purkinje cells during development, but is expressed by all Purkinje cells in the adult cerebellum. Tfap2B^+^ neurons, on the other hand, are only detected in the ML and IGL (Figures 1J,L). Thus, due to their overlapping expression pattern, we compared the expression of Tfap2A and Tfap2B with cell-type specific molecular markers in the adult cerebellum. We show that Tfap2A is found in stellate, basket and Purkinje cells, which are all labeled by parvalbumin (Figures 2A–C). Tfap2A is also found in Golgi cells, which are labeled by mGluR2 (Figures 2D–F). Tfap2B, on the other hand, is expressed only by stellate, basket and Golgi cells (Figures 2J–O). Neither Tfap2A nor Tfap2B is observed in NeuN^+^ granule cells (Figures 2G–I,P–R). Additionally, analysis of the DCN also shows that Tfap2A, but not Tfap2B, labels small GABAergic neurons in all three nuclei (Supplementary Figure S2). Thus, analysis of the temporal and spatial expression pattern of Tfap2A and Tfap2B indicates that they are expressed as early as E12.5 in the cerebellum when GABAergic neurons are produced, and that their expression persists into adulthood.

### Molecular Distinctions between Tfap2A^+^ and Tfap2B^+^ Neurons in the Developing Cerebellum

**Figure 3:**
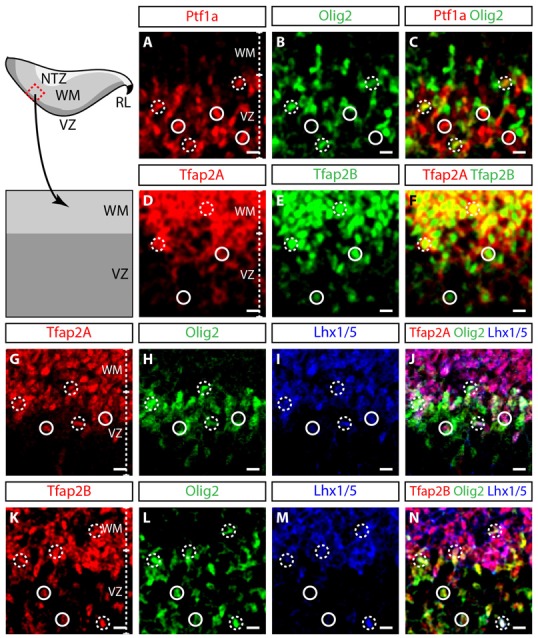
Tfap2A and Tfap2B are expressed in GABAergic precursors. **(A–C)** At E12.5, Ptf1a (red, **A**) and Olig2 (green, **B**) expression is predominantly localized in the VZ. Overlapping Ptf1a and Olig2 expression is observed in the intermediary region of the VZ (broken circle). **(D–F)** Tfap2A (red, **D**) and Tfap2B (green, **E**) are coexpressed in the VZ and WM. **(G–J)** Expression of Tfap2A (red) colocalizes with Olig2 (green) and Lhx1/5 (blue) in the VZ (broken circle). However, not all Olig2-expressing cells in the VZ express Tfap2A (continuous circle, **J**). **(K–N)** The expression of Tfap2B also colocalizes with Olig2 and Lhx1/5 (broken circle). In addition, all Olig2-expressing cells express Tfap2B (circle). Analysis performed on cerebellar vermal region of sagittal sections. Broken circles indicate cells with colocalized expression, continuous circles indicate no colocalization. Abbreviations: VZ, ventricular zone; WM, white matter. Scale bar = 10 μm.

To define the identity of Tfap2A^+^ and Tfap2B^+^ cells in the early developing cerebellum, we differentiated regions of the embryonic cerebellum with relevant molecular markers of GABAergic lineage at E12.5. The VZ can be demarcated by Ptf1a, a transcription factor responsible for differentiation of neural precursors into GABAergic neuron precursors (Hoshino et al., 2005; Yamada et al., 2014). The cerebellar VZ can also be marked by two other transcription factors namely Olig2 and Pax2 which contribute to Purkinje cell and interneuron development (Seto et al., 2014a,b; Ju et al., 2016). We observed an overlap in the expression of Ptf1a and Olig2 which demonstrates that Olig2-expressing cells are GABAergic precursors (Figures 3A–C). Therefore, Tfap2A and Tfap2B are primarily expressed by cells in the VZ and WM (Figures 3D–F). However, we observed some Tfap2B^+^ cells which do not express Tfap2A in the VZ (Figures 3D–F). Thus, the expression pattern at E12.5 indicates that Tfap2A^+^ cells consist of a subset of Tfap2B^+^ cells and suggests that their expression defines distinct neuronal subpopulations.

To determine whether Tfap2A and Tfap2B are expressed by mitotic or postmitotic GABAergic neurons, we assessed whether these cells express transcription factors Lhx1/5 (postmitotic), Olig2 and Pax2 (both mitotic and postmitotic; Maricich and Herrup, 1999; Chizhikov et al., 2006; Morales and Hatten, 2006; Zhao et al., 2007). In the VZ, most Tfap2A^+^ cells express both Olig2 and Lhx1/5, indicating that these cells in the VZ are early postmitotic GABAergic neurons (Figures 3G–J). In contrast, some Tfap2B^+^ cells in the VZ express Olig2 while others express both Olig2 and Lhx1/5 (Figures 3K–N). Thus, ~50% of Olig2-expressing cells express Tfap2A while ~100% of Olig2-expressing cells express Tfap2B (Figures 3G–N; data not shown). Together, these numbers suggest that Tfap2B^+^ cells in the VZ consist of both mitotic as well as early postmitotic GABAergic cells. Tfap2A^+^ and Tfap2B^+^ cells in the WM only express Lhx1/5 indicating that they are postmitotic GABAergic neurons (Figures 3G–N). These results show that Tfap2A^+^ and Tfap2B^+^ cells at E12.5 are GABAergic, and Tfap2A labels postmitotic cells while Tfap2B labels both mitotic and postmitotic cells in the VZ.

### Hierarchical Expression of Ptf1a, Tfap2A/B and Pax2 in the Developing Cerebellum

**Figure 4:**
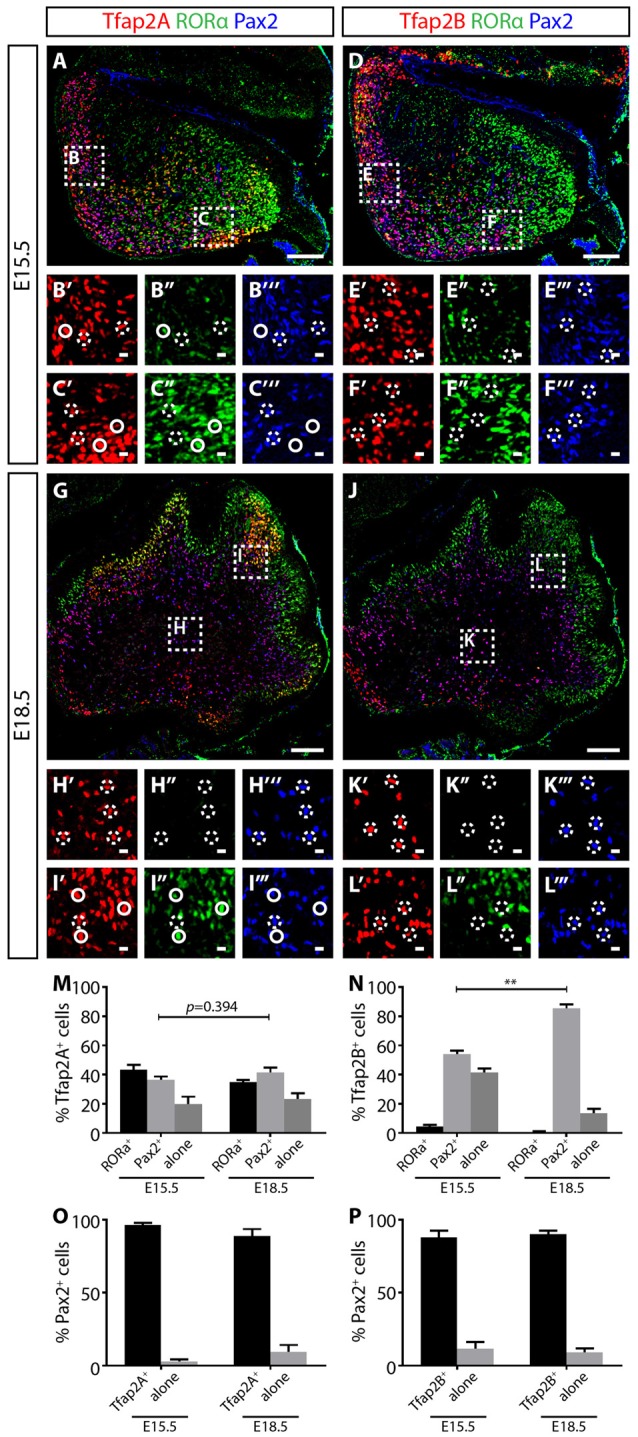
Tfap2A and Tfap2B expression precedes Pax2 in the developing cerebellum. **(A–F″′)** At E15.5, Tfap2A is expressed by a subset of RORα^+^ Purkinje cells (green) and Pax2^+^ interneurons (blue) **(A)** while Tfap2B is expressed by only Pax2^+^ interneurons **(D)**. Expression of RORα is localized mostly at the dorsal region of the cerebellum **(B′–B″′, E′–E″′)** while the expression of Pax2 is localized near the ventral region **(C′–C″′,F′–F″′)**. **(G–L″′)** At E18.5, the same Tfap2A^+^ and Tfap2B^+^ subpopulations persist, but with different distributions. **(M)** Quantification of three Tfap2A^+^ neuronal subpopulations (E15.5: Tfap2A^+^/RORα^+^ = 43 ± 3%, Tfap2A^+^/Pax2^+^ = 37 ± 2%, Tfap2A^+^-alone = 20 ± 5%; E18.5: Tfap2A^+^/RORα^+^ = 35 ± 1%, Tfap2A^+^/Pax2^+^ = 42 ± 3%, Tfap2A^+^-alone = 23 ± 4%). **(N)** Quantification of two Tfap2B^+^ neuronal subpopulations (E15.5: Tfap2B^+^/Pax2^+^ = 54 ± 3%, Tfap2B^+^-alone = 42 ± 3%; E18.5: Tfap2B^+^/Pax2^+^ = 86 ± 3%, Tfap2B^+^-alone = 14 ± 3%). **(O,P)** Analysis of Pax2^+^ neuronal population indicates that most Pax2^+^ interneurons express Tfap2A (E15.5: 97 ± 1%, E18.5: 89 ± 4%) and Tfap2B (E15.5: 88 ± 4%, E18.5: 90 ± 2%) at both E15.5 and E18.5. Analysis performed on cerebellar vermal region of sagittal sections. Continuous circles indicate colocalization of Tfap2 with RORα and broken circles indicate colocalization of Tfap2 with Pax2. Values represent mean ± SEM, *n* = 3 mice, 2 sections per mouse, *p ≤ 0.05, **p ≤ 0.01. Scale bar = 100 μm **(A,D,G,J)**, 10 μm (cropped images).

The expression of Tfap2A and Tfap2B is not observed in the VZ delineated by Ptf1a, but is expressed together with Olig2, suggesting Ptf1a expression precedes Tfap2A and Tfap2B (Figures 3G–N; Hoshino et al., 2005). In addition, Tfap2A^+^ and Tfap2B^+^ cells also overlap with Pax2 indicating that these cells identify for interneuron precursors (Supplementary Figure S3). To determine the temporal expression pattern of Tfap2A and Tfap2B in relation to cerebellar neuronal markers, we compared their expression at E15.5 and E18.5 with Pax2 and RORα, molecular markers for GABAergic interneuron precursors and postmitotic Purkinje cells, respectively (Nakagawa et al., 1997; Maricich and Herrup, 1999; Ino, 2004; Weisheit et al., 2006). Purkinje cells are specified in the VZ while interneurons diversifies later in the WM (Miale and Sidman, 1961; Palay and Chan-Palay, 1974; Leto et al., 2006, 2009). At E15.5, RORα expression is localized in the dorsal region and spreads under the external germinal layer (EGL; Figures 4A,C,D,F). On the other hand, Pax2 expression is more limited to the ventral region (Figures 4A,B,D,E). In E18.5 cerebellum, RORα is expressed in the PCL while Pax2 expression is restricted to the WM (Figures 4H,I,K,L). This pattern of expression defines the migrating status of Purkinje cells and GABAergic interneuron precursors from the VZ to the WM and finally to the cerebellar cortex providing us with the basis for analysis of Tfap2A and Tfap2B expression during this process.

At both E15.5 and E18.5, we identified three Tfap2A subpopulations (Tfap2A^+^/Pax2^+^, Tfap2A^+^/RORα^+^ and Tfap2A^+^-alone) consisting of Purkinje cells and interneurons (Figures 4A–C,G–I). At these stages, we also identified two Tfap2B subpopulations (Tfap2B^+^/Pax2^+^ and Tfap2B^+^-alone) made up of only interneurons, but not Purkinje cells, consistent with the lack of Tfap2B expression in Purkinje cells in the adult cerebellum (Figures 4D–F,J–L, see also Figures 2J–L). To examine the expression dynamics of Tfap2A and Tfap2B subpopulations, we analyzed sagittal sections of the cerebellum and examined the changes in the distribution of each subpopulation. Between E15.5 to E18.5, we detected a 5% increase in Tfap2A^+^/Pax2^+^ cells and a 3% increase in cells expressing only Tfap2A, although these changes are not statistically significant (*p* = 0.394; Figure 4M). We observed an increase in Tfap2B^+^/Pax2^+^ cells from 54% to 86% and a decrease in cells expressing only Tfap2B from 42% to 14% (*p* ≤ 0.01; Figure 4N). These changes suggests that cells previously expressing only Tfap2A or Tfap2B are now also expressing Pax2, and that, perhaps, Tfap2A^+^ and Tfap2B^+^ cells become GABAergic interneurons expressing Pax2 as they mature in the WM. Analysis of changes in Pax2 expression between E15.8 and E18.5 revealed that ~92% of Pax2^+^ cells express Tfap2A and ~89% express Tfap2B, indicating majority of Pax2^+^ interneurons express Tfap2A and Tfap2B (Figures 4O,P). Together, these results provide evidence that the expression of Tfap2A and Tfap2B precedes the expression of Pax2 and raise the possibility that Tfap2A and Tfap2B regulate the expression of Pax2.

### Manipulation of the Expression of Tfap2A and Tfap2B in GABAergic Precursors

**Figure 5:**
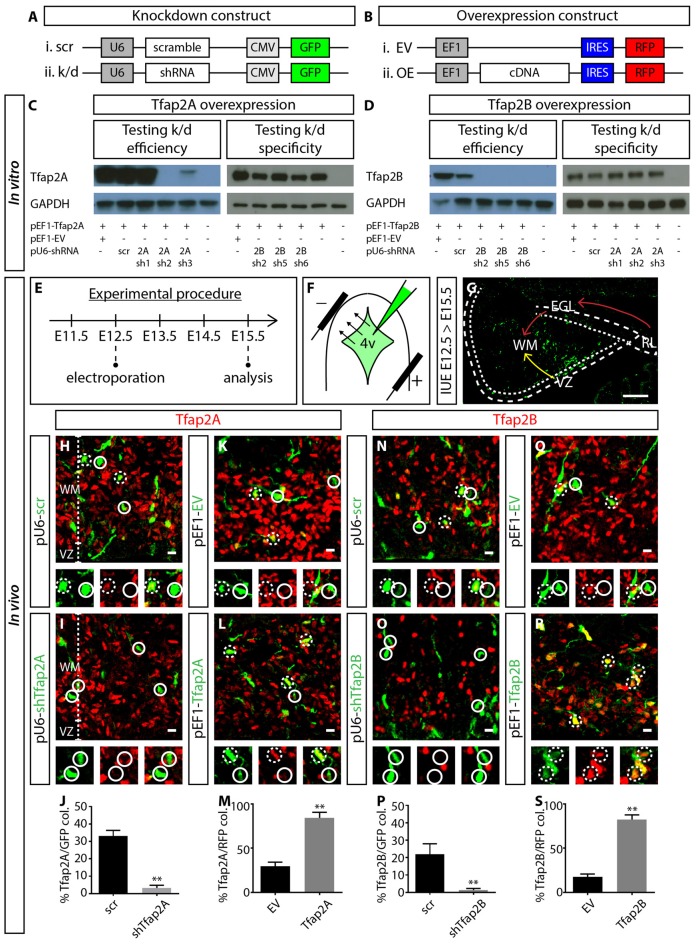
Manipulating the expression of Tfap2A and Tfap2B. **(A,B)** Plasmid constructs used to manipulate the expression of Tfap2A and Tfap2B. **(C,D)** Testing OE and knockdown constructs *in vitro* in human embryonic kidney 293T (HEK-293T) cells. Co-transfection revealed that 2Ash2 and 2Bsh2 are the most efficient (left) and specific (right) in knocking down Tfap2A **(C)** and Tfap2B **(D)** expression respectively. **(E–G)** Strategy for manipulating Tfap2A and Tfap2B expression *in utero* and experimental timeline. Transfected cells originating from the VZ migrate into the WM (yellow arrow, **G**) while cells originating from the RL migrate tangentially along the external germinal layer (EGL) before descending into the WM (red arrow, **G**). **(H–M)** Tfap2A-knockdown abolishes Tfap2A expression (3 ± 2% vs. 33 ± 3% in controls, *p* ≤ 0.01; **(H–J)** control: *n* = 3 mice, 115 cells in 11 sections; 2A k/d: *n* = 3 mice, 187 cells in 14 sections) while ectopic expression of Tfap2A results in an increase in Tfap2A^+^ cells (84 ± 6% vs. 30 ± 4% in controls, *p* ≤ 0.01; **(K–M)** control: *n* = 3 mice, 372 cells in 17 sections; 2A OE: *n* = 3 mice, 276 cells in 12 sections). **(N–S)** Similarly Tfap2B-knockdown abolishes Tfap2B expression (1 ± 1% vs. 22 ± 6% in controls, *p* ≤ 0.01; **(N–P)** control: *n* = 3 mice, 121 cells in 11 sections; 2B k/d: *n* = 3 mice, 192 cells in 12 sections) while ectopic expression of Tfap2B results in an increase in Tfap2B^+^ cells (82 ± 5% vs. 18 ± 3% in controls, *p* ≤ 0.01; **(Q–S)** control: *n* = 3 mice, 276 cells in 12 sections; 2B OE: *n* = 3 mice, 366 cells in 13 sections). Continuous circles indicate a lack of colocalization and broken circles indicate colocalization of transfected cells with respective markers. Abbreviations: 4v, 4th ventricle; EGL, external germinal layer; EV, empty vector; k/d, knockdown; OE, overexpression; RL, rhombic lip; WM, scr, scramble; white matter; VZ, ventricle zone. Values represent the mean ± SEM, *p ≤ 0.05, **p ≤ 0.01. Scale bar = 100 μm **(G)**, 10 μm **(H,I,K,L,N,O,Q,R)**.

To investigate the functional relevance of Tfap2A and Tfap2B during the development of GABAergic neuronal subtypes, we set out to manipulate the expression of Tfap2A and Tfap2B via IUE. We first generated knockdown constructs containing short hairpin RNA (shRNA) under mouse *U6* promoter and GFP under *CMV* promoter (p*U6*-shRNA-*CMV*-GFP; Figure 5A). We also generated cDNA constructs expressing either *Tfap2A* or *Tfap2B* and red fluorescent protein (RFP) separated by Internal Ribosomal Entry Site (IRES) sequence under the *elongation factor 1* (*EF1*) promoter (p*EF1*-cDNA-IRES-RFP; Figure 5B). To determine the efficiency and specificity of these constructs, knockdown and OE plasmids were co-transfected into HEK-293T cells which do not normally express either of these transcription factors. Following that, western blot was carried out to identify the most efficient and specific shRNA that targets the respective member of Tfap2. We found that pU6-2Ash2 and pU6-2Bsh2 to be most efficient and specific in targeting Tfap2A and Tfap2B expression, respectively, so they were subsequently used for *in vivo* experiments (Figures 5C,D).

For IUE experiments, plasmid constructs were delivered into the fourth ventricle (4v) of E12.5 wild-type (WT) embryos. Cells lining the 4v, which includes the VZ and RL with sparse Tfap2A and Tfap2B expression, were transfected via electroporation (Figures 5E,F, Supplementary Figure S4). After 3 days, transfected neurons from the VZ migrate anteriorly into the WM while neurons originating from the RL migrate tangentially over the cerebellar surface before descending into the WM (Figure 5G). Therefore, we analyzed the identity of transfected cells which have migrated into the WM. In control or scrambled experiments, ~33% of GFP^+^ cells express Tfap2A while ~22% express Tfap2B (Figures 5H,J,N,P). In knockdown experiments, Tfap2A knockdown reduces the percentage of transfected cells expressing Tfap2A by 10-fold while Tfap2B knockdown reduces the percentage of transfected cells expressing Tfap2B by 20-fold (Figures 5H–J,N–P). In the OE experiments, we observed a ~three-fold and five-fold increase in the number of transfected cells expressing Tfap2A and Tfap2B, respectively (Figures 5K–M,Q–S). This strategy provides means to manipulate Tfap2 expression *in vivo* through suppression and induction of their expression.

### Consequences of Manipulating Tfap2A and Tfap2B Expression on Specification of GABAergic Interneurons

**Figure 6:**
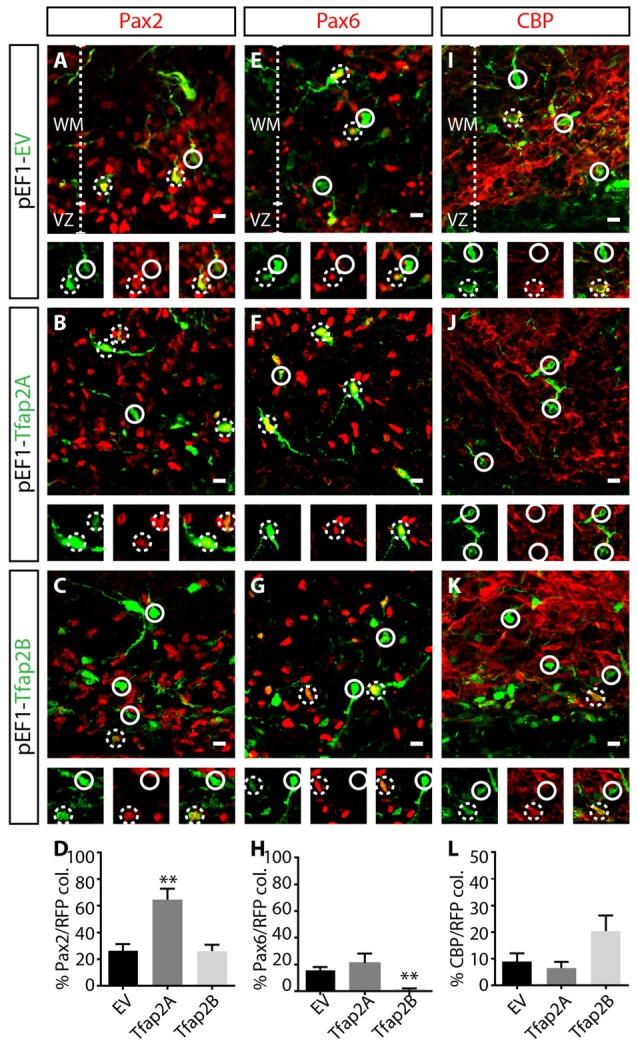
Tfap2A misexpression results in an increase in Pax2 expression, while Tfap2B misexpression results in a decrease in the expression of Pax6. **(A–L)** Consequences of Tfap2A and Tfap2B misexpression on generation of cerebellar neuronal subpopulations. Misexpression of Tfap2A results in an increase in the number of Pax2^+^ cells (54 ± 11% vs. 26 ± 5% in controls, *p* ≤ 0.01; **(A,B,D)** control: *n* = 3 mice, 347 cells in 13 sections; 2A OE: *n* = 3 mice, 130 cells in 14 sections), but misexpression of Tfap2B did not change the number of Pax2^+^ cells compared to controls (24 ± 6% vs. 26 ± 5% in controls, *p* = 0.88; **(B–D)** control: *n* = 3 mice, 347 cells in 12 sections; 2B OE: *n* = 3 mice, 212 cells in 12 sections). Misexpression of Tfap2B, but not Tfap2A, results in a decrease in the number of Pax6^+^ cells (Tfap2B: 1 ± 1% vs. 16 ± 3% in controls, *p* ≤ 0.01; **(F–H)** Tfap2A: 19 ± 8% vs. 16 ± 3% in controls, *p* = 0.66; **(E,G,H)** control: *n* = 3 mice, 542 cells in 14 sections; 2A OE: *n* = 3 mice, 65 cells in 8 sections; 2B OE: *n* = 3 mice, 337 cells in 13 sections). Misexpression of Tfap2A and Tfap2B has no impact on the number of CBP^+^ Purkinje cells (Tfap2A: 6 ± 2% vs. 9 ± 3% in controls, *p* = 0.85; **(I,K,L)** Tfap2B: 20 ± 6% vs. 9 ± 3% in controls, *p* = 0.063; **(J–L)** control: *n* = 3 mice, 365 cells in 15 sections; 2A OE: *n* = 3 mice 160 cells in 10 sections; 2B OE: *n* = 261 cells in 9 sections). Continuous circles indicate lack of colocalization and broken circles indicate colocalization of transfected cells with respective markers. Abbreviations: VZ, ventricular zone; WM, white matter. Values represent the mean ± SEM, *p ≤ 0.05, **p ≤ 0.01. Scale bar = 10 μm.

Ptf1a is a major determinant of specification of GABAergic neuronal fate (Pascual et al., 2007; Hori and Hoshino, 2012; Yamada et al., 2014), but determinants of GABAergic interneurons vs. GABAergic projection neurons are limited. To assess the capability of Tfap2A and Tfap2B in directing the specification of GABAergic neuronal subtypes, we analyzed consequences of misexpressing each transcription factor on the expression of neuronal markers, Pax2, Pax6 and CBP which correspond to GABAergic interneurons, glutamatergic neurons and Purkinje cells, respectively. We first examined whether misexpression of Tfap2A or Tfap2B is sufficient to regulate Pax2 expression. We did not observe any changes in the number of Pax2^+^ interneurons in Tfap2B-misexpressed cells compared to control (Figures 6A,C,D). Interestingly, we observed a ~16% decrease in the number of Pax6^+^ excitatory cells within Tfap2B-misexpressed cells compared to control (Figures 6E,G,H). Next, we assessed consequences of misexpressing Tfap2A. Despite the inability of Tfap2B to change the number of Pax2^+^ interneurons, we found a two-fold increase in the number of Pax2^+^ interneurons in Tfap2A-misexpressed cells relative to control in the WM (Figures 6A,B,D). No change was observed in the number of Pax6^+^ RL-derived cells within Tfap2A-misexpressed cells (Figures 6E,F,H). Additionally, misexpression of Tfap2A or Tfap2B had no impact on the expression of CBP (Figures 6I–L). Together we show that the expression of Tfap2A, but not Tfap2B, has the ability to direct specification of Pax2^+^ GABAergic interneurons. Conversely, the expression of Tfap2B, but not Tfap2A can suppress the expression of Pax6.

**Figure 7:**
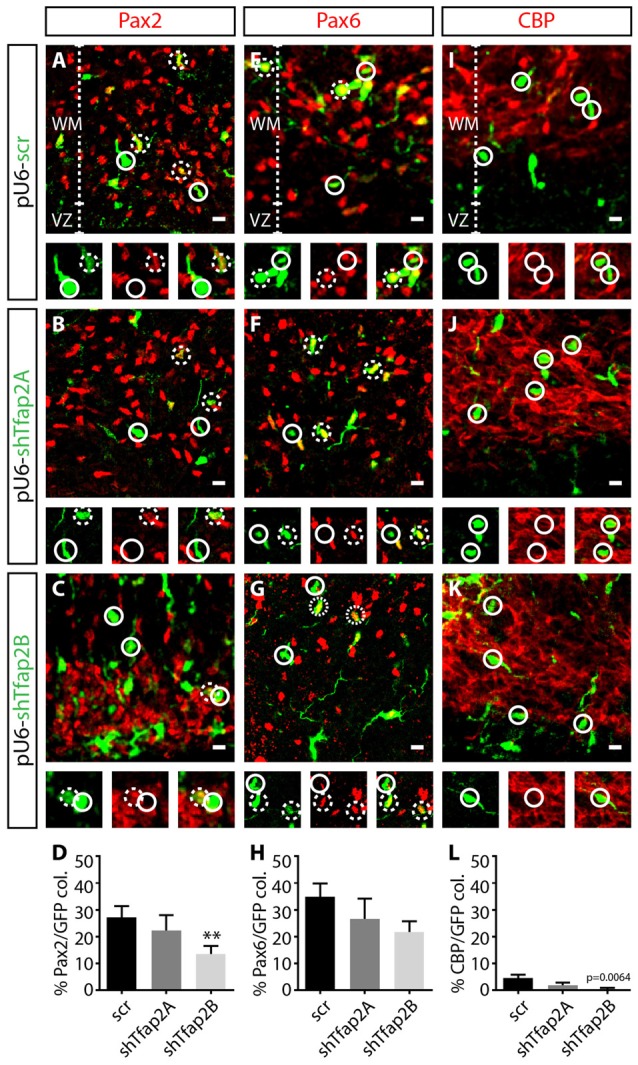
Knockdown of the expression of Tfap2B, but not Tfap2A, results in a reduction in Pax2 expression. **(A–H)** Knockdown of Tfap2A expression in the VZ does not affect the number of Pax2^+^ cells (22 ± 6% vs. 27 ± 4% in controls, *p* = 0.53; **(A,B,D)** control: *n* = 3 mice, 153 cells in 12 sections; 2A k/d: *n* = 3 mice, 87 cells in 14 sections) or number of Pax6^+^ cells compared to controls (27 ± 8% vs. 34 ± 4% in controls, *p* = 0.29; **(E,F,H)** control: *n* = 3 mice, 241 cells in 13 sections; 2A k/d: *n* = 3 mice, 188 cells in 13 sections). However, knockdown of Tfap2B expression results in a significant decrease in number of Pax2^+^ cells (13 ± 3% vs. 27 ± 4% in controls, *p* ≤ 0.01; **(A,C,D)** control: *n* = 3 mice, 153 cells in 12 sections; 2B k/d: *n* = 3 mice, 391 cells in 26 sections), without affecting the number of Pax6^+^ cells (22 ± 4% vs. 34 ± 4% in controls, *p* = 0.054; **(E,G,H)** control: *n* = 3 mice, 241 cells in 13 sections; 2B k/d: *n* = 3 mice, 211 cells in 12 sections). **(I–L)** A scant percentage of CBP^+^ Purkinje cells are transfected, although knockdown of Tfap2B, but not Tfap2A, slightly decreased this subpopulation (Tfap2B: 0.5 ± 0.5% vs. 4 ± 1% in controls, *p* = 0.0064; **(J–L)** Tfap2A: 2 ± 1% vs. 4 ± 1% in controls, *p* = 0.067; **(I,J,L)** control: *n* = 3 mice, 307 cells in 13 sections; 2A k/d: *n* = 3 mice, 206 cells in 14 sections; 2B k/d: *n* = 3 mice, 253 cells in 13 sections). Continuous circles indicate lack of colocalization and broken circles indicate colocalization of transfected cells with respective markers. Abbreviations: VZ, ventricular zone; WM, white matter. Values represent the mean ± SEM, *p ≤ 0.05, **p ≤ 0.01. Scale bar = 10 μm.

To determine the requirements of Tfap2 for specification of GABAergic neurons, we assessed the effects of knocking down the expression of Tfap2A or Tfap2B on the expression of Pax2, Pax6 and CBP at E15.5. We found a ~15% reduction in the number of Pax2^+^ interneurons in Tfap2B-knockdown cells in the WM (Figures 7A,C,D). In contrast, no change in number of Pax2^+^ interneurons were observed in Tfap2A-knockdown cells (Figures 7A,B,D). Knockdown of Tfap2A or Tfap2B had no impact on the expression of Pax6 as predicted (Figures 7E–H). Knockdown of Tfap2B results in a modest reduction in the number of CBP^+^ Purkinje cells, although Purkinje cells do not typically express Tfap2B (Figures 2, 7J–L). No change in number of CBP^+^ cells was detected following Tfap2A knockdown (Figures 7I,K,L). Moreover, because Purkinje cells are scarcely transfected at E12.5 (Figure 7L), our results suggest that Tfap2A and Tfap2B are unable to influence CBP expression. Knockdown of Tfap2B in the VZ of the developing cerebellum at E12.5 results in a decrease of Pax2 expression, indicating that Tfap2B is necessary for the specification of cerebellar GABAergic interneurons. Together, our results demonstrate that Tfap2A and Tfap2B have independent roles in directing the diversification of cerebellar GABAergic neuronal subtypes in the cerebellum. Moreover, our results indicate that only Tfap2B is indispensable for the specification of cerebellar GABAergic interneurons.

## Discussion

**Figure 8:**
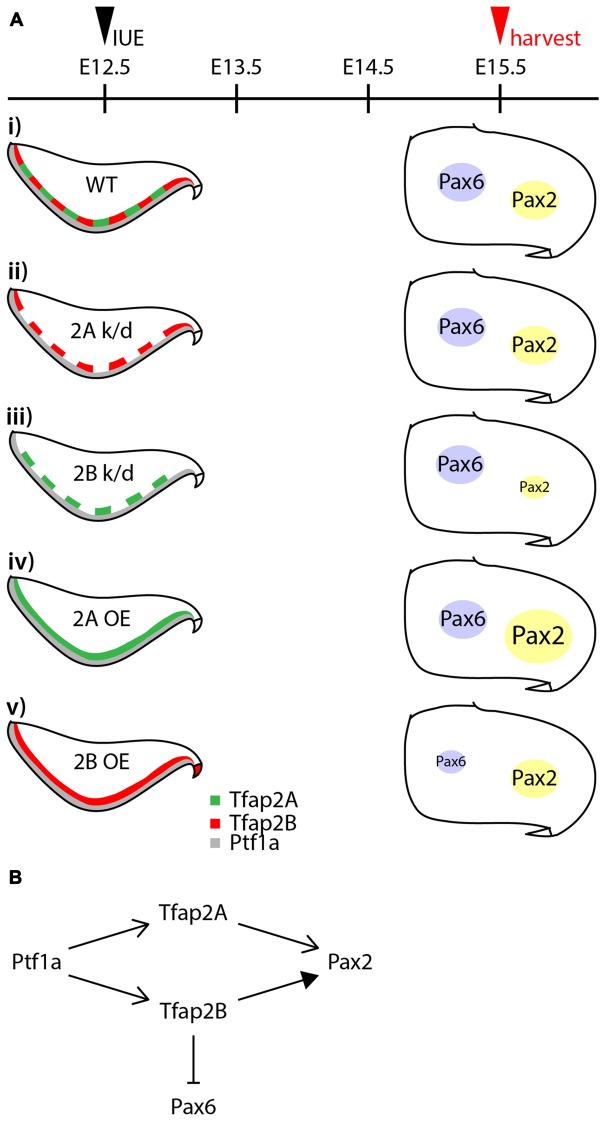
Summary of the activities of Tfap2A and Tfap2B in GABAergic interneuron specification. **(A)** Summary of results from knocking down and ectopic expression of Tfap2A and Tfap2B. **(Ai)** In a wild-type, expression of Tfap2A and Tfap2B occur in an overlapping manner in the ventricular zone at E12.5 which is observed superior to Ptf1a territory. Some expression of Pax6 and Pax2 is observed in the white matter of the developing cerebellum at E15.5. Knockdown of Tfap2A does not show any changes in the expression of Pax2 or Pax6 **(Aii)**. Knockdown of Tfap2B results in a decrease in Pax2 expression **(Aiii)**. Ectopic expression of Tfap2A results in an increase in Pax2 expression **(Aiv)**, while ectopic expression of Tfap2B results in a decrease in Pax6 expression **(Av)**. **(B)** Proposed activities of Tfap2A and Tfap2B in the development of cerebellar neurons. Expression of Tfap2A and Tfap2B downstream of Ptf1a play distinct roles to ensure generation of Pax2^+^ interneurons. Tfap2A may also function to produce Purkinje cells, but at earlier developmental stages. In addition to generation of GABAergic interneurons, Tfap2B plays a role in the suppression of Pax6 expression. Abbreviations: IUE, *in utero* electroporation; k/d, knockdown; OE, overexpression; RL, rhombic lip; VZ, ventricular zone; WT, wild-type.

GABAergic neurons in the cerebellar cortex are subdivided into projection neurons and interneurons, consisting of at least four subtypes derived from the same pool of multipotent progenitors (Hoshino et al., 2005; Pascual et al., 2007; Yamada et al., 2014), but the transcriptional program that directs this diversification is not well defined. To explore determinants of the specification of GABAergic neuron progenitors into GABAergic neuronal subtypes, we examined the expression and function of Tfap2 family of transcription factors (summarized in Figure 8). We discuss how the differential expression of Tfap2A and Tfap2B defines GABAergic neuronal subtypes and their contribution to the generation and specification of cerebellar GABAergic neurons.

### Combinatorial Expression of Tfap2 Transcription Factors and Cerebellar GABAergic Neuronal Subtypes

There is emerging evidence that GABAergic neuronal subtypes are defined by their unique expression of transcription factors (Helms and Johnson, 2003; Wonders and Anderson, 2006; Hoshino, 2012; Achim et al., 2014). In the cortex, for instance, GABAergic interneurons are derived from Nkx2.1 domain of the medial ganglionic eminence and ventral caudal ganglionic eminence (Cobos et al., 2006; Wonders and Anderson, 2006). Subsequently, GABAergic neuronal subtypes can be differentiated by their distinct combinatorial expression of Dlx1/2, Dlx5/6 and Lhx6 (Cobos et al., 2006; Wonders and Anderson, 2006). In more caudal regions of the nervous system, the differential expression of Lbx1, Lhx1/5, Pax2, Gsh1/2, Dbx2 and GATA2/3 defines GABAergic interneuron subtypes in the spinal cord; Lhx1/5 is expressed by all GABAergic neurons and Pax2 is expressed only by interneurons in the cerebellum (Helms and Johnson, 2003; Hori and Hoshino, 2012). Additionally, Ptf1a is expressed by all early GABAergic precursors in both the cerebellum and spinal cord (Hori and Hoshino, 2012). Thus, in comparison to the cortex and spinal cord, there are less distinguishing molecular features for labeling and monitoring of GABAergic neuronal subtypes in the cerebellum.

We show in this study that the combinatorial expression of Tfap2A and Tfap2B may distinguish GABAergic projection neurons from interneurons in the developing cerebellum. In the adult cerebellum, Tfap2A is expressed in all GABAergic neurons, whereas Tfap2B is selectively expressed in the interneuronal population (Figure 2). In contrast to Ptf1a and Olig2, whose expression is extinguished by E13.5 (Pascual et al., 2007; Seto et al., 2014a; Ju et al., 2016), the expression of Tfap2 transcription factors persists into adulthood. However, Tfap2B might be transiently expressed in early Purkinje cells during embryonic development (Figure 3). Our discovery that Tfap2 transcription factors are dynamically and, perhaps, differentially expressed by GABAergic neuronal subtypes in the cerebellum, provides a novel set of molecular markers that can complement a growing list of genetic tools for future characterization and lineage analysis of these neurons. There is now a number of mouse lines for the tagging and monitoring of cerebellar GABAergic neurons: *Gad67*::GFP and *Ptf1a*::Cre label all GABAergic neurons (Yamanaka et al., 2004; Pascual et al., 2007; Yamada et al., 2014), *Corl2*::GFP and *Olig2*::Cre mark projection neurons (Nakatani et al., 2014; Seto et al., 2014b; Ju et al., 2016) and *Pax2*::GFP selectively labels GABAergic interneurons (Leto et al., 2006; Weisheit et al., 2006). The emergence of these tools will permit a systematic examination of the development, function as well as molecular profile of cerebellar GABAergic neuronal subtypes.

### Distinct Roles of Tfap2A and Tfap2B in Establishment of GABAergic Neuronal Subtype Identity

Tfap2 family of transcription factors regulates a myriad of developmental processes in the nervous system, kidney, skeleton, limbs, skin and eye (Schorle et al., 1996; Zhang et al., 1996; Moser et al., 1997; Nottoli et al., 1998; West-Mays et al., 1999; Eckert et al., 2005; Seberg et al., 2017). Members of this family exhibit functional redundancy in zebrafish. For instance, Tfap2A and Tfap2E are redundant in their regulation of melanophore development (Van Otterloo et al., 2010). Additionally, Tfap2A and Tfap2C are redundant in their induction of neural crest development (Hoffman et al., 2007; Li and Cornell, 2007). In the mouse, however, members of this family appear to play more discrete roles in developmental processes. For example, Tfap2A is indispensable for craniofacial, eye, and limb development (Schorle et al., 1996; Nottoli et al., 1998); and, Tfap2B is indispensable for kidney development (Moser et al., 1997). Consistently, our results indicate that Tfap2A and Tfap2B play independent roles in the specification of GABAergic interneurons during mouse cerebellar development.

There is growing evidence that, in addition to Ptf1a, Tfap2A and Tfap2B play a crucial role in the specification of GABAergic neuronal subtypes (Figures 6, 7). The expression of Ptf1a in neural precursors directs generation of GABAergic neurons and suppresses generation of glutamatergic neurons in the cerebellum, spinal cord and retina (Glasgow et al., 2005; Hoshino et al., 2005; Nakhai et al., 2007; Pascual et al., 2007). Tfap2A and Tfap2B expression is dependent on Ptf1a in the retina (Jin et al., 2015). Both transcription factors have the ability to promote generation of GABAergic/glycinergic amacrine cells and concomitantly suppress the generation of excitatory photoreceptors and bipolar cells (Jin et al., 2015). In this study, we show that following determination of GABAergic fate of neural precursors in the VZ by Ptf1a, Tfap2A and Tfap2B subsequently serve to ensure generation of GABAergic neurons and suppression of excitatory neurons. Tfap2A alone has the ability to promote generation of GABAergic interneurons while Tfap2B, but not Tfap2A, can suppress the generation of excitatory cells. Thus, Tfap2A and Tfap2B appear to have distinct roles in the specification of GABAergic/glycinergic neurons in the cerebellum, and may serve to delegate the activities of Ptf1a similar to the function of Tfap2A/B in the retina (Figure 8B).

Distinguishing molecular features of GABAergic interneurons vs. GABAergic projection neurons in the cerebellum have not be well defined. A set of bHLH transcription factors Ascl1 and Ngn1 may be involved in specification of interneurons and projection neurons, respectively. Ascl1 is required for generation of Pax2^+^ interneurons, but not Purkinje cells, whereas Ngn1 is required for generation of Purkinje cells, but not Pax2^+^ interneurons (Grimaldi et al., 2009; Lundell et al., 2009). Lim homeodomain transcription factors Lhx1 and Lhx5 and their cofactor Ldb1 are required for production of Purkinje cells, but not Pax2^+^ interneurons (Zordan et al., 2008). Additionally, the transcription factor Corl2 plays a role in maturation of Purkinje cells (Wang et al., 2011). Besides Ascl1, cerebellar GABAergic interneurons are dependent on cyclin D2 and cell-cycle dynamics (Huard et al., 1999; Leto et al., 2011). In this study, we add to this list members of Tfap2 family of transcription factors whose expression defines cerebellar GABAergic projection vs. interneurons, and acts to ensure specification of GABAergic interneurons, but not projection neurons.

## Author Contributions

NZ and AIC designed the studies and prepared the manuscript with comments from all authors. NZ performed all the experiments and analyzed the data. SPK carried out *in utero* electroporation experiments and revised the manuscript. ART carried out analysis in the DCN.

## Conflict of Interest Statement

The authors declare that the research was conducted in the absence of any commercial or financial relationships that could be construed as a potential conflict of interest.

## Supplementary Material

FIGURE S1 Tfap2A is selectively expressed in the Purkinje cell layer during development but is uniformly expressed in the adult cerebellum. **(A–D)** Colocalization of Tfap2A (red, **B′,C′,D′,B″′,C″′,D″′**) and CBP (green, **B″,C″,D″,B″′,C″′,D″′**) at early post-natal day 7 show selective Purkinje cell expression in the developing cerebellum. **(E–H)** The expression of Tfap2A (red, **F′,G′,H′,F″′,G″′,H″′**) and PVA (green, **F″,G″,H″,F″′,G″′,H″′**) in Purkinje cells from different regions of the cerebellum does not show a medio-lateral distribution pattern. Analysis was performed on coronal sections of the cerebellum from P60 mice. Broken circles indicate cells with colocalized expression, continuous circles indicate no colocalization. Abbreviations: CBP, calbindin; DCN, deep cerebellar nuclei; IGL, internal granular layer ML, molecular layer; PCL, Purkinje cell layer; PVA, parvalbumin. Scale bar = 1000 μm **(A,E)**, 20 μm **(B′–D″′,F′–H″′)**.

FIGURE S2 Tfap2A, but not Tfap2B, is expressed in GABAergic neurons in the DCN. **(A–F)** Expression of Tfap2A (green, **A–C**) is restricted to small GABAergic neurons of the DCN, marked by MAP2 (red) and Gad67 (blue). Tfap2B expression, on the other hand, is absent in all three nuclei in the DCN. Scale bar = 10 μm.

FIGURE S3 Tfap2A and Tfap2B are expressed by GABAergic interneuron precursors in the embryonic cerebellum. **(A–C)** Delineation of the E12.5 cerebellum with Ptf1a (red, **A**), a molecular marker that labels the ventricular zone, and Pax2 (green, **B**), a GABAergic interneuron precursor marker. **(D–I)** A subset of Tfap2A **(D)** and Tfap2B **(G)** colocalizes with Pax2 interneuron marker in the embryonic cerebellum. Abbreviations: VZ, ventricular zone; WM, white matter. Scale bar = 10 μm.

FIGURE S4 *In utero* electroporation of the cerebellum at E12.5 transfects cells of the ventricular zone and rhombic lip. **(A,B)** Summary of targeted regions and strategy for *in utero* electroporation. **(C–I)** Schematic diagram of the expression of respective molecular markers in the embryonic cerebellum at E13.5. **(C′–E″)** GABAergic molecular markers, Ptf1a and Olig2, label the ventricular zone while Pax2 labels the white matter layer. **(F′–G″)** Glutamatergic molecular markers, Pax6 and calretinin, label transfected cells that arise from the rhombic lip. **(H′–I″)** Tfap2A and Tfap2B cells are mostly found in the white matter layer which are preferentially targeted during *in utero* electroporation. Abbreviations: NTZ, nuclear transitory zone; RL, rhombic lip; WM, white matter; VZ, ventricular zone. Scale bar = 10 μm.
