# Supplementary figures and images for: Distinct Activities of Tfap2A and Tfap2B in the Specification of GABAergic Interneurons in the Developing Cerebellum
Source: Front Mol Neurosci. 2017 Aug 31;10:281. doi: 10.3389/fnmol.2017.00281 (PMC5583517; doi:10.3389/fnmol.2017.00281)

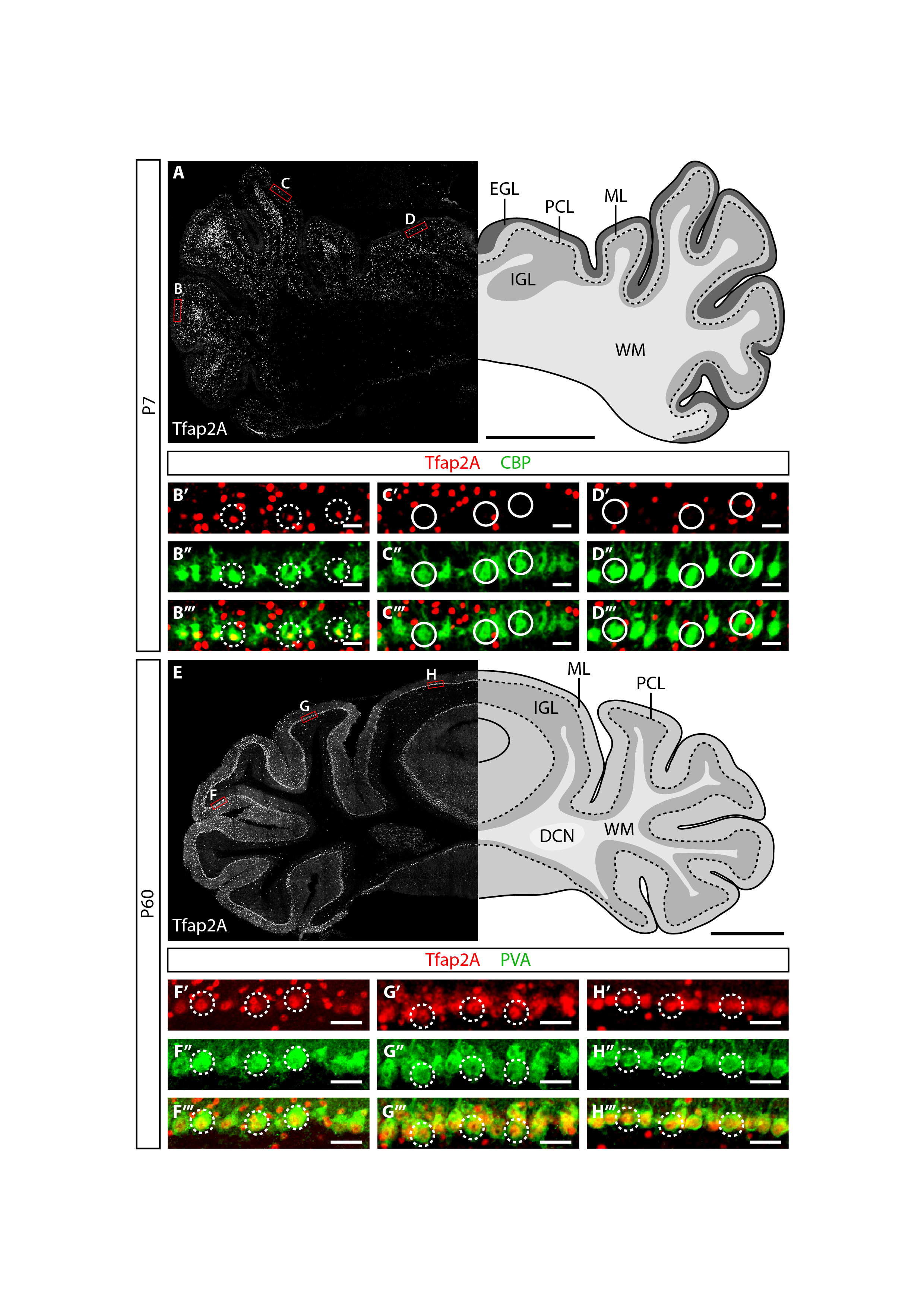

Supplement: FIGURE S1 — Tfap2A is selectively expressed in the Purkinje cell layer during development but is uniformly expressed in the adult cerebellum. (A–D) Colocalization of Tfap2A (red, B′,C′,D′,B″′,C″′,D″′) and CBP (green, B″,C″,D″,B″′,C″′,D″′) at early post-natal day 7 show selective Purkinje cell expression in the developing cerebellum. (E–H) The expression of Tfap2A (red, F′,G′,H′,F″′,G″′,H″′) and PVA (green, F″,G″,H″,F″′,G″′,H″′) in Purkinje cells from different regions of the cerebellum does not show a medio-lateral distribution pattern. Analysis was performed on coronal sections of the cerebellum from P60 mice. Broken circles indicate cells with colocalized expression, continuous circles indicate no colocalization. Abbreviations: CBP, calbindin; DCN, deep cerebellar nuclei; IGL, internal granular layer ML, molecular layer; PCL, Purkinje cell layer; PVA, parvalbumin. Scale bar = 1000 μm (A,E), 20 μm (B′–D″′,F′–H″′). [file Image_1.jpeg]

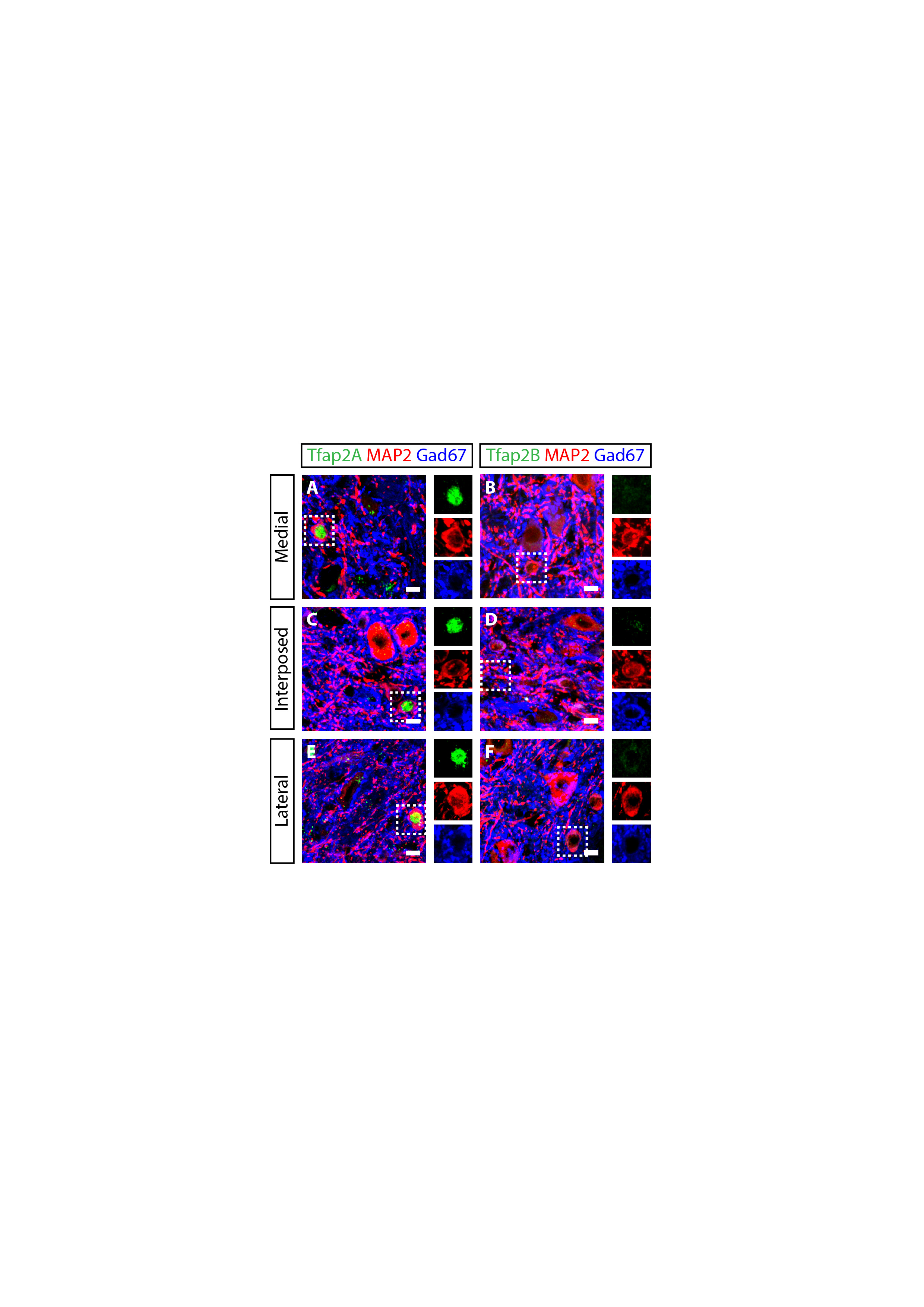

Supplement: FIGURE S2 — Tfap2A, but not Tfap2B, is expressed in GABAergic neurons in the DCN. (A–F) Expression of Tfap2A (green, A–C) is restricted to small GABAergic neurons of the DCN, marked by MAP2 (red) and Gad67 (blue). Tfap2B expression, on the other hand, is absent in all three nuclei in the DCN. Scale bar = 10 μm. [file Image_2.jpeg]

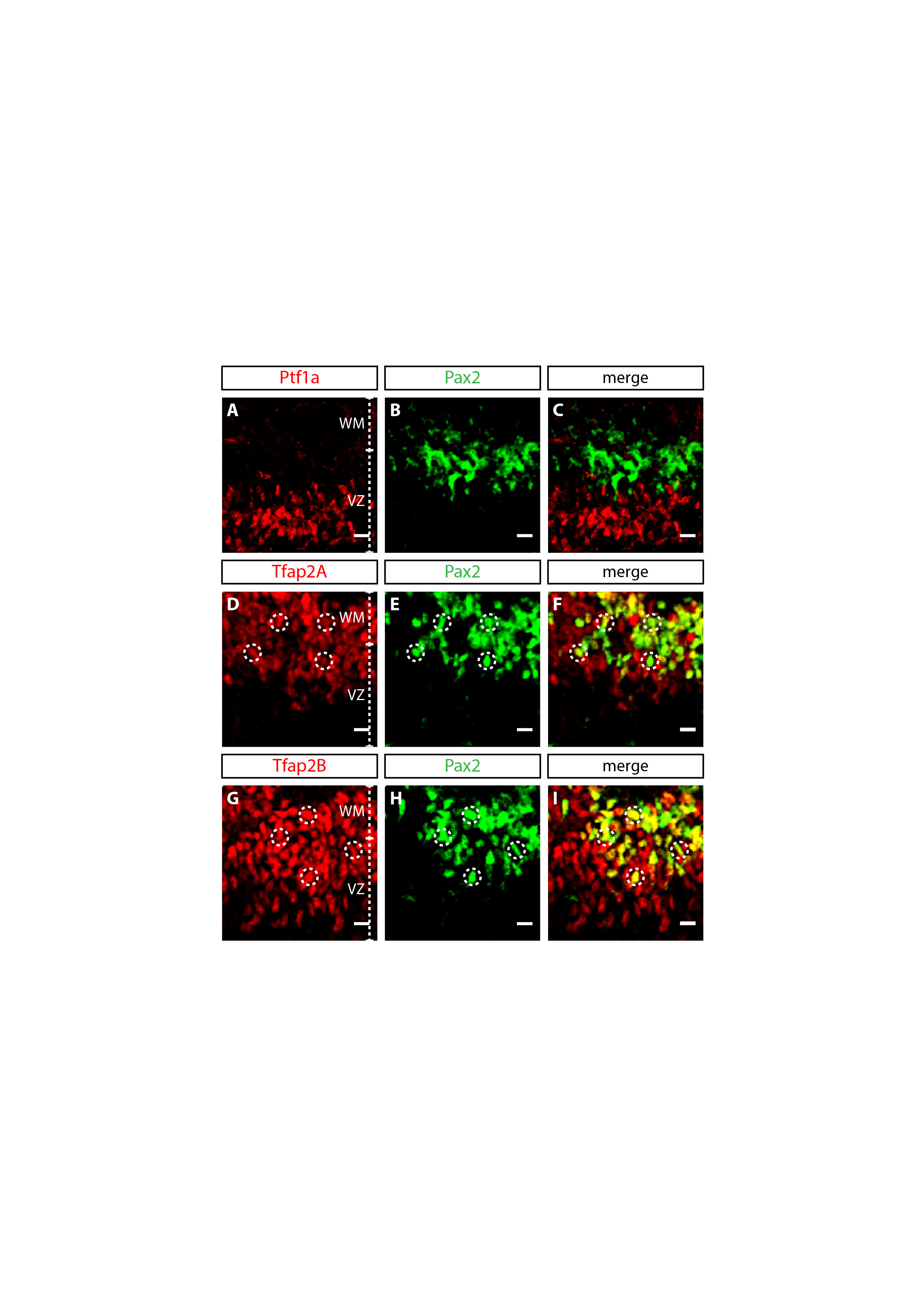

Supplement: FIGURE S3 — Tfap2A and Tfap2B are expressed by GABAergic interneuron precursors in the embryonic cerebellum. (A–C) Delineation of the E12.5 cerebellum with Ptf1a (red, A), a molecular marker that labels the ventricular zone, and Pax2 (green, B), a GABAergic interneuron precursor marker. (D–I) A subset of Tfap2A (D) and Tfap2B (G) colocalizes with Pax2 interneuron marker in the embryonic cerebellum. Abbreviations: VZ, ventricular zone; WM, white matter. Scale bar = 10 μm. [file Image_3.jpeg]

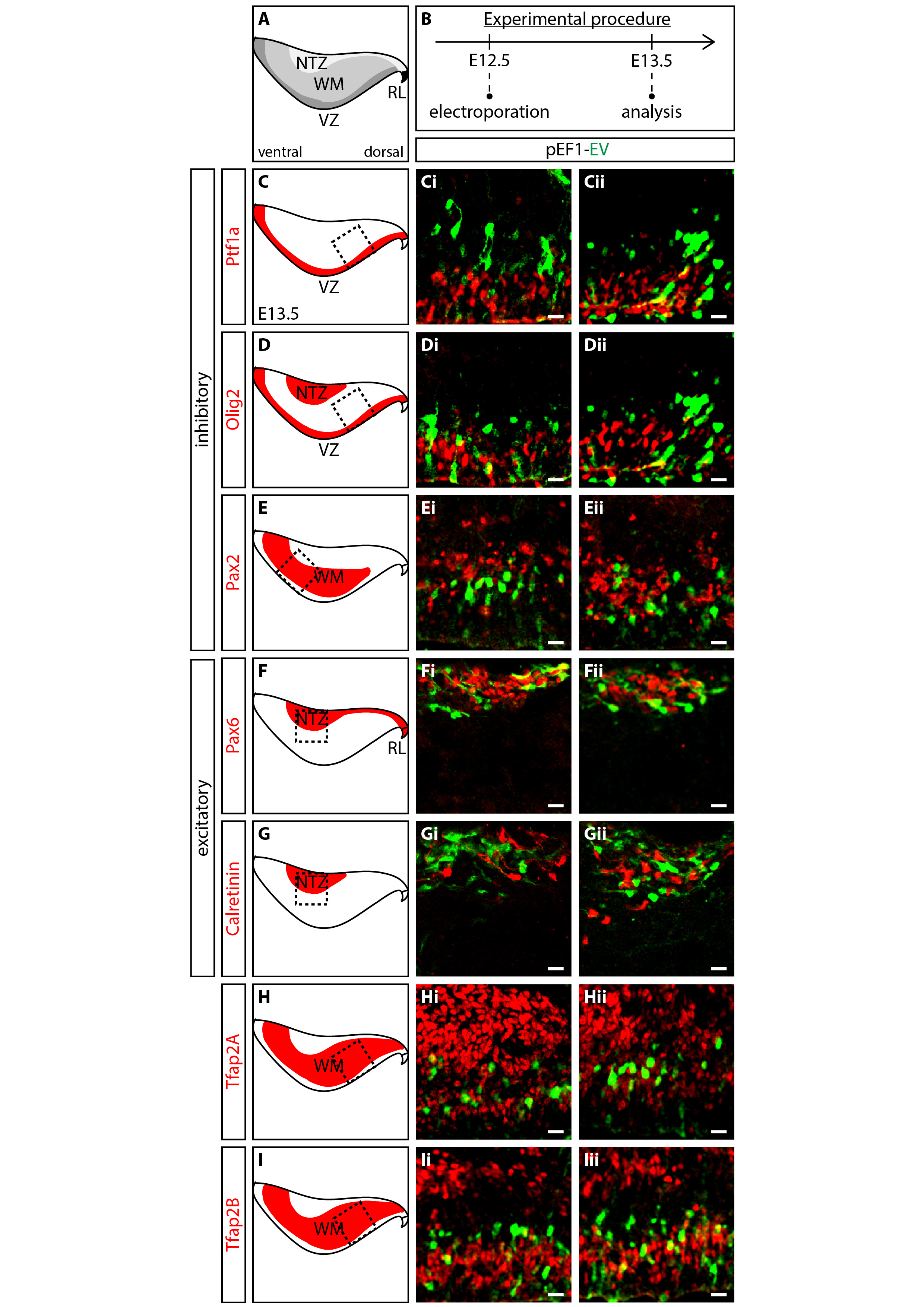

Supplement: FIGURE S4 — In utero electroporation of the cerebellum at E12.5 transfects cells of the ventricular zone and rhombic lip. (A,B) Summary of targeted regions and strategy for in utero electroporation. (C–I) Schematic diagram of the expression of respective molecular markers in the embryonic cerebellum at E13.5. (C′–E″) GABAergic molecular markers, Ptf1a and Olig2, label the ventricular zone while Pax2 labels the white matter layer. (F′–G″) Glutamatergic molecular markers, Pax6 and calretinin, label transfected cells that arise from the rhombic lip. (H′–I″) Tfap2A and Tfap2B cells are mostly found in the white matter layer which are preferentially targeted during in utero electroporation. Abbreviations: NTZ, nuclear transitory zone; RL, rhombic lip; WM, white matter; VZ, ventricular zone. Scale bar = 10 μm. [file Image_4.jpeg]
